# Structural Empowerment of Nurses in the Hospital Setting: A Cross-Sectional Study

**DOI:** 10.3390/nursrep15120444

**Published:** 2025-12-11

**Authors:** Marlene Ribeiro, Diana Sanches, Sónia Barros, Mariana Gonçalves, Susana Castro, Tânia Oliveira, Renata Gasparino, Olga Ribeiro

**Affiliations:** 1 ICBAS, Abel Salazar Institute of Biomedical Sciences, University of Porto, 4050-313 Porto, Portugal; susanafcastro@ipoporto.min-saude.pt (S.C.); taniaoliveira@ulsaave.min-saude.pt (T.O.); 2 RISE-Health, Faculty of Medicine, University of Porto, 4200-319 Porto, Portugal; olgaribeiro@esenf.pt; 3 Tâmega and Sousa Local Health Unit, 4560-136 Penafiel, Portugal; 4 Gaia and Espinho Local Health Unit, 4434-502 Vila Nova de Gaia, Portugal; diana.sanches@ulsge.min-saude.pt; 5 São João Local Health Unit, 4200-319 Porto, Portugal; sonia.barros@ulssjoao.min-saude.pt; 6 Department of Community Medicine, Information and Health Decision Sciences (MEDCIDS), Faculty of Medicine, University of Porto, 4099-002 Porto, Portugal; 7 Lusíadas Lisboa Hospital, 1500-458 Lisboa, Portugal; mariana.mendes.goncalves@lusiadas.pt; 8 National School of Public Health, NOVA University Lisbon, 1600-560 Lisboa, Portugal; 9 Portuguese Oncology Institute of Porto Francisco Gentil, 4200-072 Porto, Portugal; 10 Oncology Nursing Research Unit IPO Porto Research Center (CI-IPOP), Portuguese Oncology Institute of Porto (IPO Porto)/Porto Comprehensive Cancer Centre (Porto.CCC) & RISE@CI-IPOP (Health Rsearch Network), 4200-072 Porto, Portugal; 11 Alto Ave Local Health Unit, 4835-044 Guimarães, Portugal; 12 School of Nursing, Universidade Estadual de Campinas—Unicamp, Campinas 13083-887, São Paulo, Brazil; grenata@unicamp.br; 13 Nursing School of Porto, 4200-072 Porto, Portugal

**Keywords:** empowerment, hospitals, nurses, nurse specialists, workplace environments

## Abstract

**Background/Objectives**: Structural empowerment involves access to opportunities, information, support, and resources within the work environment. These elements are crucial as they foster nurses’ professional growth and autonomy. Given their significance, understanding hospital nurses’ perceptions of structural empowerment is important. Therefore, the aim of this study is to explore hospital nurses’ perceptions of their levels of structural empowerment. **Methods**: This cross-sectional study included 684 nurses from a Portuguese hospital, conducted between November 2024 and January 2025. The questionnaire collected demographic data and employed the Conditions of Work Effectiveness Questionnaire II. **Results**: The mean total score for structural empowerment was 18.74 (SD = 3.46), with significant differences found between nurses and specialist nurses, for those with the specialist title (*p* = 0.0477) and within the professional category (*p* = 0.0058), as well as between nurses working day shifts and those working day and night shifts (*p* < 0.0001). Additional significant differences were observed between nurses from the Medicine department (median [Q1–Q3]: 19.25 [17.00–21.50]) and those from the Surgery department (18.17 [16.00–20.25], *p* = 0.0104), as well as between Generation Z nurses (19.58 [17.33–21.83]) and Generation Y nurses (18.29 [16.25–20.33], *p* = 0.0018). **Conclusions**: These results underscore the importance of consistently promoting structural empowerment across the nursing staff, addressing disparities between groups, and improving both professional development and quality of patient care.

## 1. Introduction

Work environments in the healthcare sector have become increasingly demanding and complex as a result of advances in science and health technologies, as well as the citizen demand for safe and quality care. Also in nursing, the advances and evolution of the profession have inevitably led to new demands in the settings where professional practice takes place [1].

The International Council of Nurses (ICN) has been issuing recommendations emphasising that nursing practice environments should adopt innovative policy frameworks that enhance nurses’ well-being, professional satisfaction, safety, quality of care, and organisational performance [2].

Several studies identify the practice environment as a variable that influences nursing care outcomes, where better professional nursing environments are linked to lower burnout levels, higher nurse satisfaction [3,4,5,6], improved perceptions of care quality, a reduced intention to leave the organisation, and increased productivity [7,8,9,10]. Likewise, consistent with scientific evidence from recent decades, a favourable nursing practice environment significantly affects the quality and safety of patient care, as well as the effectiveness of health services, organisations, and systems [11,12].

The nursing practice environments that foster the best outcomes for nurses, patients, and organisations are characterised by a set of key attributes, including leadership, autonomy, and structural empowerment, exemplary professional practice, collaborative work, engagement, and shared decision-making, among others [1,13]. In a study by Travis and Fitzpatrick [14], most nurses working in magnet hospitals, hospitals characterised by these attributes, reported high levels of structural empowerment. Furthermore, these environments were associated with greater nurse work effectiveness and improved care delivery, leading the authors to conclude that empowering work environments should be promoted [1,13].

The concept of structural empowerment stems from Kanter’s work on power within organisations [15], where the author identified organisational structures that promote employee effectiveness, motivation, and productivity. Using this framework, Kanter defined the environment of structural empowerment [15].

Structural empowerment refers to an organisation’s ability to provide employees with access to the resources necessary to support the performance of their roles. It relates to the specific social conditions and policies within the work environment that facilitate access to opportunities, information, support, and resources, considering the characteristics of formal or informal powers [16,17,18].

According to Laschinger [18], access to opportunities involves the potential for growth within the organisation and the chance to acquire more knowledge and skills. Access to resources refers to the ability to obtain all necessary and appropriate means to perform the work. Access to information encompasses access to the formal and informal knowledge needed to be effective in the workplace and to understand organisational policies and decisions. Access to support involves receiving guidance and assistance from subordinates, colleagues, and superiors [18].

Access to empowerment structures is strengthened by formal and informal power. Formal power relates to work activities and their perceived significance for organisational goals, as well as the employee’s visibility, opportunities for creative work, and autonomy in decision-making. Informal power is linked to relationships and alliances at all levels of the organisation [18].

Evidence shows that better outcomes in structural empowerment are associated with more positive results for patients, professionals, and organisations, such as increased motivation and job satisfaction [19,20,21,22], organisational citizenship behaviour [23], organisational commitment [16,24], participation in decision-making processes and autonomy [25,26], reduction in stress and burnout [16,27]. Furthermore, it has a positive effect on the delivery of high-quality care, work effectiveness, and the improvement of safety culture [16,28]. Empowered nurses can apply more complex and specialised skills, both in decision-making and problem-solving related to care provision, such as offering innovative solutions [29,30].

In this way, for nurses to perform their duties effectively, it is essential that health organisations adopt management models and policies that enhance the empowerment of these professionals [31].

Despite the extensive international evidence, there is a lack of national studies examining structural empowerment among nurses in hospital settings. This gap limits the adoption of organisational strategies tailored to the national context that could foster a supportive environment for nursing practice. Therefore, this study aimed to assess the levels of structural empowerment among nurses in the hospital setting.

## 2. Materials and Methods

### 2.1. Design and Setting

This was a cross-sectional study, conducted in accordance with the Strengthening the Reporting of Observational Studies in Epidemiology (STROBE) Statement [32], which took place between November 2024 and January 2025 at a university hospital in northern Portugal.

This hospital is a national reference in health education and research, central and a leader in several medical and surgical specialities for various districts in northern Portugal. It has approximately 1100 inpatient beds, a 24-h emergency service for adults, paediatrics, and obstetrics, and the most specialised capacity to provide care to all critically ill patients in its various intensive care units. By the end of 2023, the number of employees was approximately 6675 professionals, of whom 2580 were nurses [33].

### 2.2. Participants

The potential participants in this study were all nurses, regardless of their contractual status, working in the departments of Medicine, Surgery, Intensive Care, Emergency, and Women and Children’s Health. All nurses with at least six months of professional experience at the hospital were included. Those who were inactive due to medical certificates or leave were excluded.

### 2.3. Instruments

The questionnaire served as the data collection instrument and was divided into two sections.

In the initial section, a set of closed-answer questions was provided to characterise the sample, including queries about gender, age, education, professional title awarded by the Portuguese Nurses’ Order, nursing speciality area, professional category within the institution, work department, shift type worked, and length of professional experience, both overall and within the specific context.

In the second section, the Portuguese version of the Conditions of Work Effectiveness Questionnaire II (CWEQ–II) was used [34]. This instrument measures structural empowerment. It was created by Laschinger et al. [35] and contains 19 items divided into six dimensions: Opportunity (3 items), Resources (3 items), Information (3 items), Support (3 items), Formal Power (3 items), and Informal Power (4 items). The response options follow a 5-point Likert scale, with Opportunity, Formal Power, and Informal Power scores ranging from 1 (None) to 5 (Very Much); Information scores from 1 (No Knowledge) to 5 (Very Much Knowledge); and Support and Resources scores from 1 (None) to 5 (Very Much).

In the Opportunity, Resources, Information, and Support dimensions, the average score of each is calculated by summing the item scores and dividing by the number of items. These scores can range from 1 to 5, with higher scores indicating greater access to opportunities, resources, information, and support. An overall structural empowerment score is obtained by summing these four dimensions, resulting in a range from 4 to 20. Higher scores reflect stronger perceptions of working in an empowering environment [18]. Regarding the Formal Power and Informal Power dimensions, scores also range from 1 to 5, with higher scores representing work activities that confer greater formal or positional power relative to formal power, and stronger alliance and networks in the organisation relative to informal power [18]. The sum of the six dimensions’ averages constitutes the total score, which spans from 6 to 30 [18,34]. The following cut-off points for the 6-dimensional scale are set: between 6 and 13 are classified as low levels of structural empowerment, from 14 to 22 as moderate levels of structural empowerment, and from 23 to 30 as high levels of structural empowerment [18].

A measure of Global Empowerment (GE) serves as a validation index. The GE score is calculated by summing and averaging the two global empowerment items at the end of the questionnaire and can range from 1 to 5. Higher scores indicate stronger perceptions of working in an empowered workplace [18].

The CWEQ–II was cross-culturally adapted and validated for the Portuguese population by Teixeira et al. [34], demonstrating strong psychometric properties. It is important to note that the overall Cronbach’s alpha coefficient was 0.911, with values ranging from 0.678 to 0.889 across the respective dimensions (Opportunity = 0.854, Resources = 0.797, Information = 0.859, Support = 0.889, Formal Power = 0.811, and Informal Power = 0.678) [34].

### 2.4. Data Collection

Data collection began after obtaining favourable ethical and legal approvals. This was achieved through an initial meeting at each department, where the study, its objectives, and all other relevant information were presented. The nurse manager of each department, or another designated member, collaborated in verifying inclusion and exclusion criteria and distributing and collecting the questionnaires, which were completed on paper in a sealed envelope.

Additionally, two meetings were held at each department to clarify any questions and distribute and collect the questionnaires.

Participants completed the questionnaires individually during their work shift at a location of their choice within the department. To reduce social desirability bias, managers or other designated individuals responsible for distributing and collecting the sealed envelopes were not present during the completion and had no involvement beyond that task. Additionally, participants were instructed to complete the questionnaire independently and without consulting colleagues, maintaining anonymity and confidentiality throughout.

During the data collection period, no significant organisational changes (such as restructurings, staff fluctuations, or management transitions) were reported that could have influenced nurses’ perception of structural empowerment.

### 2.5. Data Analysis

Initially, the data were organised and checked using Microsoft Excel^®^ (Office 365) version 2501.

Descriptive methods were employed to characterise the variables, and data distribution was assessed using the Shapiro–Wilk test.

As the quantitative variables did not follow a normal distribution, Spearman’s correlation coefficient was used to examine correlations between them [36]. This coefficient ranges from −1 to 1, with values closer to −1 indicating a negative or inverse relationship, values nearer to 1 indicating a positive relationship, and values close to 0 suggesting no correlation. Cohen [37] recommends the following classification of correlation coefficients: 0.10 to 0.29 (weak), 0.30 to 0.49 (moderate), and greater than or equal to 0.50 (strong).

Non-parametric tests were used for group comparisons. The Mann–Whitney U test was employed to compare CWEQ-II scores between groups defined by categorical variables with two categories [36]. When the categorical variable included more than two categories, the Kruskal–Wallis test was applied, followed by Dunn’s post-hoc test [36].

Internal consistency of the CWEQ-II in the study sample was evaluated using Cronbach’s alpha [38]. This coefficient ranges from 0 to 1, with values greater than 0.7 indicating reliability between measures [39].

All analyses were conducted using Statistical Analysis System^®^ version 9.4 and IBM SPSS Statistics version 23 statistical software, with a significance level of 5%.

### 2.6. Ethical Approval and Informed Consent

The study was conducted in accordance with the ethical principles of the Declaration of Helsinki, as well as with the guiding principles of good practice in scientific research involving human participants.

Thus, the study protocol was reviewed by the hospital’s health ethics committee and the data protection officer, both of whom provided a favourable opinion. It was then approved at a meeting of the board of directors.

Potential participants were informed about the research aims and the voluntary nature of their involvement, along with the option to withdraw at any point without any consequences. This information was also given in writing through a Participant Information Letter. The Informed Consent form was utilised, and all participants were given ample time to decide whether to participate.

Furthermore, no data that could identify individual participants was collected, and all questionnaires and data were anonymised and coded.

## 3. Results

1200 questionnaires were made available, and 699 were collected, yielding a response rate of 58.25%. However, 15 questionnaires were excluded due to incomplete responses, resulting in a final sample of 684 participants for analysis.

The sample mainly consisted of female participants (80.85%) who were Generation Y or Millennials (60.23%), with an average age of 40.34 years (SD = 9.41). Most participants identified as nurses in the professional category (76.90%); however, 251 nurses held the title of specialist nurses (36.70%). The most common departments were Medicine and Surgery (32.60% and 29.68%, respectively). All their sociodemographic, educational, and professional characteristics are shown in Table 1.

Regarding the levels of structural empowerment, the average total score was 18.74 (SD = 3.46), with the Opportunity dimension achieving the highest score, averaging 3.89 (SD = 0.81), followed by the Informal Power dimension with an average of 3.35 (SD = 0.70). The Formal Power dimension recorded the lowest score, with an average of 2.57 (SD = 0.83), followed by the Resources dimension with an average of 2.82 (SD = 0.80). All results related to structural empowerment and its dimensions are detailed in Table 2.

The correlations between CWEQ–II total score and the time of professional practice, as well as between CWEQ–II total score and the time of professional practice within the department, were analysed using Spearman’s correlation coefficient. No statistically significant relationships were observed in any of the tests conducted (*r* = 0.0131, *p* = 0.7325; *r* = −0.0582, *p* = 0.1281, respectively).

Comparisons were conducted between groups regarding the total structural empowerment score. The Mann–Whitney U test showed that the total score for structural empowerment was significantly higher among nurses with a specialist title granted by the Portuguese Nurses’ Order (median [Q1–Q3]: 19.17 [16.75–21.17]) compared to those without a specialist title (median [Q1–Q3]: 18.42 [16.42–20.42]), as evidenced by a *p*-value of 0.0477. Similarly, in the professional category, nurses formally integrated within the specialist nurse category exhibited higher levels of total structural empowerment (median [Q1–Q3]: 19.33 [17.42–21.25]) compared to those in the general nurse category (median [Q1–Q3]: 18.42 [16.33–20.50]), with a *p*-value of 0.0058. Moreover, nurses working solely day shifts reported significantly higher levels of total structural empowerment (median [Q1–Q3]: 19.58 [17.50–22.00]) than those working both day and night shifts (median [Q1–Q3]: 18.33 [16.17–20.33]), with a *p*-value < 0.0001.

The Kruskal–Wallis test revealed statistically significant differences between nurses of different generations (*p* = 0.0018) and various work departments (*p* = 0.0104). The results of all comparisons are presented in Table 3.

Considering the results from the Kruskal–Wallis test, Dunn’s post-hoc test was used to compare the groups two by two (multiple comparisons) and to determine where the difference was statistically significant (Table 4).

The total structural empowerment score was significantly higher among nurses in the Medicine department (median [Q1–Q3]: 19.25 [17.00–21.50]) compared to nurses in the Surgery department (median [Q1–Q3]: 18.17 [16.00–20.25]), as indicated by a *p*-value of 0.039 in Dunn’s post-hoc test. It was also significantly higher among Generation Z nurses (median [Q1–Q3]: 19.58 [17.33–21.83]) compared to Generation Y or Millennial nurses (median [Q1–Q3]: 18.29 [16.25–20.33]), with a *p*-value of 0.004 in Dunn’s post-hoc test (Table 4).

Cronbach’s α values for all dimensions were above 0.70, indicating acceptable internal consistency: Opportunity (0.84), Information (0.91), Support (0.92), Resources (0.84), Formal Power (0.80), and Informal Power (0.75).

## 4. Discussion

The primary aim of this study was to investigate nurses’ perceptions of structural empowerment in the hospital setting. 684 nurses from the Medicine, Surgery, Intensive Care, Emergency, and Women’s and Children’s Health departments participated in the research, most of whom were from Generation Y or Millennials, who now comprise the largest nursing workforce in these settings [40].

Participants reported a mean total score of 18.74 (SD = 3.46), corresponding to moderate levels of structural empowerment. The Opportunity dimension scored the highest, at an average of 3.89 (SD = 0.81), followed by the Informal Power dimension at 3.35 (SD = 0.70). Similar results were found in the study by Teixeira et al. [41], conducted at another hospital in northern Portugal, where 354 nurses reported an average of 18.6 (SD = 3.3) for structural empowerment. The Opportunity dimension also had the highest score (3.6; SD = 0.8), followed by the Informal Power dimension (3.5; SD = 0.7) [41]. It should also be noted that, in both studies, the Formal Power dimension received the lowest scores, with an average of 2.57 (SD = 0.83) in the present study and 2.5 (SD = 0.8) in the study by Teixeira et al. [41], followed by the Resources dimension with an average of 2.82 (SD = 0.80) in the present study and 2.7 (SD = 0.8) in the Teixeira et al. study [41].

The consistency between the results reported in both studies suggests a shared perception of structural empowerment among nurses in the hospital setting in northern Portugal. Small differences in mean values may reflect contextual variations, such as organisational structure or the timing of data collection.

The consistently lower scores on the Formal Power dimension, observed in both, given the items’ content, may indicate a perception of limited recognition and rewards for nurses’ activities within organisational processes. Furthermore, the higher scores on the Informal Power dimension may reflect nurses’ perceptions of multidisciplinary collaboration and recognition of their competencies by other health professionals they work with, although these do not influence institutional decision-making and management. The discrepancy between the results in these two dimensions suggests that, although nurses hold informal power within institutions to make decisions and act autonomously in their work environment, there is an urgent need to strengthen their formal role and importance in organisational decision-making [1,13,42].

Concerning the Resources dimension, the results of this study align with those of previous studies [41], in which the dimension received lower mean scores. This result may reflect ongoing challenges in ensuring that nurses have sufficient time to deliver all necessary care and can access help when needed. Limited resources constrain clinical practice and may also lead to increased workload, job stress, and moral distress, ultimately diminishing job satisfaction and retention [16,43]. Furthermore, restricted access to resources is identified as a barrier to nurses’ autonomy and professional development, as it can limit the delivery of essential care and hinder the ability to implement evidence-based practices or innovative solutions [13,30].

When compared with international studies, although the levels of structural empowerment are also considered moderate, there are some differences between the dimensions that present the highest and lowest average scores, and this fact may be related to the local organizational culture of each work environment, as well as the social mandate of the nursing profession, which differs from the Portuguese one [30,31,44].

In the study by Travis and Fitzpatrick [14], although no statistically significant relationship was found between the origin of nurses and levels of structural empowerment, the majority of nurses were from magnet hospitals (77.7%). The levels of total structural empowerment, and across all dimensions, were high and notably higher than those reported in the present study, as well as in other studies [30,31,41,44]. The mean total score of structural empowerment was 22.80 (SD = 3.38), and the Resource dimension had the lowest mean at 3.27 (SD = 0.90). The Opportunity and Informal Power dimensions obtained the highest mean scores, at 4.17 (SD = 0.61) and 4.08 (SD = 0.65), respectively [14]. In fact, structural empowerment is considered a fundamental component that serves to structure the work environments that characterise magnet hospitals [13].

The Resources dimension consistently scores the lowest, emphasising the need for targeted organisational interventions. To address these issues, nursing managers could implement strategies such as optimising staffing levels, ensuring access to necessary materials in both quantity and quality, providing support, and allocating adequate time for clinical tasks. While these actions are vital, more comprehensive and innovative strategies can also be explored. For instance, reducing bureaucratic tasks, making work more flexible and organised by adapting and planning the workload to match service complexity, improving information systems for faster, more accurate access and better communication, implementing remote monitoring and automation tools for registration, and introducing performance recognition programmes that value efficient resource use.

Another finding when comparing this study with the study conducted by Teixeira et al. [41] is that, in both studies, no statistically significant relationships were identified between the time of professional practice and the time of professional practice within the department, with the total structural empowerment score. However, in the study of Teixeira et al. [41], there is a positive, statistically significant relationship between the total structural empowerment score and nurses’ age. In this study, the analysis was performed across generations, as this information may be relevant from a management perspective for enhancing outcomes related to professionals, which is particularly important in the current context, such as nurse satisfaction and retention [45].

Thus, the results reveal statistically significant differences in perceptions of structural empowerment across generations of nurses, especially between Generation Z (19.58 [17.33–21.83]) and Generation Y or Millennials (18.29 [16.25–20.33]), with the former showing higher median values that are statistically significant (*p* = 0.004). In this way, Generation Z expressed more positive perceptions of access to opportunities, information, resources, and support, as well as formal and informal power, compared to Generation Y or Millennials, who currently constitute most of the nursing workforce.

This finding may be related to the differences in the context of entry into the labour market. Generation Z nurses entered the workforce at a time when healthcare organisations were more participative structures with more structured integration periods, factors that may contribute to more favourable perceptions of structural empowerment, as these are two aspects that this generation values [46,47]. These data are consistent with Moon and Lee [48], suggesting the importance of a horizontal organisational culture for Generation Z nurses. 

On the other hand, Generation Y, despite valuing autonomy and professional development equally [9,49], faced, to a greater extent upon entering the labour market, practice environments characterised by budgetary restrictions and a scarcity of human resources, which may have negatively influenced their perception of empowerment. Balay-odao et al. [50] demonstrated, in a study with Saudi Arabian nurses, that Millennials enjoy working in environments where they have opportunities for career advancement, work flexibility, recognition of their performance, and a healthy work–life balance. Structural empowerment fosters a sense of autonomy, competence, and connection, motivating employees and enabling them to remain energetic and productive.

These results, according to the study by Al Sabei et al. [51], emphasise the importance of leadership and human resource management strategies in nursing, taking into account the generational profile of professionals and adopting differentiated approaches to promote structural empowerment. The implementation of mentoring programmes, the creation of opportunities for professional development and progression, and the reinforcement of various formal communication channels can contribute to standardising positive perceptions across different generations, thereby promoting not only job satisfaction but also retention and the quality of care.

Another relevant aspect that must be considered in nursing management strategies is related to promoting the professional development of nursing teams through training to differentiate and specialise skills, as well as providing opportunities for nurses to advance within the organisation, with improved working conditions and employment contracts tailored to the nurses’ skills [29].

According to the results of this study, nurses who have obtained the title of specialist in a nursing field from the Portuguese Nurses’ Order, as well as those who are formally performing these roles under an employment contract suitable for the specialist nurse professional category, exhibit statistically significant differences in empowerment levels, perceiving greater structural empowerment within organisations (*p* = 0.0477, and *p* = 0.0058, respectively).

The awarding of the title of specialist nurse by the Portuguese Nurses’ Order is a formal recognition of advanced skills in a nursing area [52]. This can impact the professional identity of these nurses and, as a result, foster an appreciation within the organisation. Although informal, this appreciation helps them perceive better access to opportunities, resources, information, and support [13,53].

When this recognition is officially complemented by an employment contract in the specialist nurse category, the environment becomes more conducive to structural empowerment because the nurse perceives that they have greater autonomy and formal legitimacy to act, lead, and influence processes [54]. Therefore, institutional policies must, in addition to encouraging the acquisition of the title, ensure it aligns with the contractual and functional status [13].

The present study also found statistically significant differences between nurses in the Medicine department (19.25 [17.00–21.50]) and those in the Surgery department (18.17 [16.00–20.50]), with the former reporting higher perceptions of structural empowerment (*p* = 0.039).

Although no studies were found that explored the differences between these two departments, some contextual factors may explain these findings. Surgical department services are often characterised by medical prescriptions linked to surgical procedures and more rigid hierarchical structures, which may strengthen the interdependent dimension of nursing care while simultaneously limiting nurses’ autonomy and formal decision-making power [55]. Conversely, in medical department services, nursing practice might be more closely associated with autonomous activities and more collaborative relationships with multidisciplinary teams [56]. These organisational features may foster more positive perceptions of empowerment among medical department nurses, particularly regarding access to opportunities, information, and informal power. However, further studies are needed to explore these differences and their possible causes. Recognising these contextual variations is essential to adapting management strategies that reinforce structural empowerment across different clinical areas.

Finally, nurses who work only day shifts report a higher perception of structural empowerment than those working both day and night shifts. This aspect has not been examined in other studies. However, in the hospital environment, some organisational dynamics may explain these results. Nurses working solely day shifts spend more time with multidisciplinary teams and hospital management decision-makers, which enhances access to information, support, and opportunities, key aspects of structural empowerment. Additionally, on rotating shifts, particularly night shifts, nurses work in smaller teams with less contact with institutional decision-makers, which can reinforce feelings of overload and a lack of support.

Overall, this study contributes significantly by enhancing the understanding of the structural empowerment of nurses in hospital settings. Alongside confirming trends identified in previous national and international research, this study investigates previously less-explored aspects, such as differences between hospital departments, shift schedules, and generational backgrounds. It presents interesting findings that can assist nursing managers and institutional decision-makers in developing strategies to improve access to resources, formal power, and support, tailored to the specific needs of various clinical areas and professional groups.

### Study Limitations

Despite the relevance of the results obtained and the contributions of this study, it has some limitations that should be acknowledged. The design of the cross-sectional study does not allow for causal conclusions to be drawn. Furthermore, although some results of this study align with those of another study conducted in a hospital context in northern Portugal, structural empowerment should be analysed in different contexts simultaneously to enable broader generalisation. Additionally, other contextual factors such as leadership, organisational climate, and institutional policies that may influence perceptions of structural empowerment were not examined.

## 5. Conclusions

This study found moderate levels of structural empowerment among nurses at a hospital in northern Portugal, with the dimensions of Opportunity and Informal Power achieving the highest scores, and those of Resources and Formal Power registering the lowest.

Significant differences were observed between some sample characterisation variables and structural empowerment. Therefore, Generation Z nurses, specialist nurses, those working during the day, and in medical department services showed a greater perception of structural empowerment.

These results emphasise the need for management strategies that promote equitable access to resources, information, and decision-making to prevent disparities within nursing teams. Future research should include other organisational variables and adopt longitudinal and mixed-methods approaches to better understand empowerment and its impact on nursing practice and outcomes for patients, nurses, and institutions.

## Figures and Tables

**Table 1:** Sociodemographic, educational, and professional characteristics of the participants (*n* = 684).

Sociodemographic, Educational, and Professional Characteristics	
Gender *n* (%)	
Female	553 (80.85)
Male	131 (19.15)
Age (years) Mean; Std. Dev.	40.34; 9.41
Generation *n* (%)	
Generation Y or Millennials	412 (60.23)
Generation X	167 (24.42)
Generation Z	86 (12.57)
Generation Baby Boomer	19 (2.78)
Education *n* (%)	
Bachelor’s degree	576 (84.21)
Master’s degree	108 (15.79)
Professional title *n* (%)	
Nurse	433 (63.30)
Specialist nurse	251 (36.70)
Nursing specialisation *n* (%)	
Medical–Surgical nursing	81 (32.27)
Rehabilitation nursing	78 (31.08)
Community nursing	35 (13.94)
Child and Pediatric Health nursing	34 (13.55)
Maternal and Obstetric nursing	23 (9.16)
Professional category *n* (%)	
Nurse	526 (76.90)
Specialist nurse	151 (22.08)
Nurse manager	7 (1.02)
Work department *n* (%)	
Medicine department	223 (32.60)
Surgery department	203 (29.68)
Intensive Care department	113 (16.52)
Woman and Child department	77 (11.26)
Emergency department	68 (9.94)
Work schedule *n* (%)	
Day and night shifts	523 (76.46)
Day shifts	161 (23.54)
Time of professional practice (years) Mean; Std. Dev.	17.02; 9.30
Time of professional practice in the department (years) Mean; Std. Dev.	10.96; 9.04

*n*—absolute frequency; %—relative frequency; Std. Dev.—Standard deviation.

**Table 2:** Characterisation of structural empowerment levels, for each CWEQ–II dimension and total score (*n* = 684).

CWEQ–II	Mean	SD	Min.	Q1	Median	Q3	Max.
CWEQ–II Opportunity	3.89	0.81	1.00	3.33	4.00	4.67	5.00
CWEQ–II Information	2.98	0.84	1.00	2.67	3.00	3.33	5.00
CWEQ–II Support	2.95	0.94	1.00	2.33	3.00	3.67	5.00
CWEQ–II Resources	2.82	0.80	1.00	2.33	3.00	3.33	5.00
CWEQ–II Structural Empowerment	12.64	2.46	5.00	11.33	12.67	14.00	20.00
CWEQ–II Formal Power	2.57	0.83	1.00	2.00	2.67	3.00	5.00
CWEQ–II Informal Power	3.35	0.70	1.00	3.00	3.50	4.00	5.00
CWEQ–II Total Score	18.74	3.46	7.00	16.50	18.75	20.75	30.00
CWEQ–II Global Empowerment (GE)	3.18	0.95	1.00	2.50	3.00	4.00	5.00

S.D.—Standard deviation; Min.—Minimum; Q1—Quartile 1; Q3—Quartile 3; Max.—Maximum.

**Table 3:** Differences in levels of structural empowerment (total score) between groups.

Variables	*n*	Mean	SD	Min.	Q1	Median	Q3	Max.	*p*-Value
Gender									
Male	131	18.54	3.86	7.00	16.33	18.50	21.00	29.08	0.5151 *
Female	553	18.78	3.36	7.00	16.67	18.75	20.75	30.00	
Generation									
Generation Z	86	19.75	3.29	13.50	17.33	19.58	21.83	28.83	**0.0018** **
Generation Y or Millennials	412	18.38	3.33	7.00	16.25	18.29	20.33	30.00	
Generation X	167	19.01	3.52	8.50	16.83	19.00	20.83	29.08	
Professional title									
Specialist nurse	251	19.98	3.59	7.00	16.75	19.17	21.17	29.00	**0.0477** *
Nurse	433	18.59	3.38	7.00	16.42	18.42	20.42	30.00	
Nursing specialization									
Maternal and Obstetric	23	17.12	3.58	8.17	14.92	17.33	19.75	22.17	0.0696 **
Child and Pediatric Health	34	18.72	3.31	11.75	16.75	19.08	20.17	29.00	
Medical–Surgical	81	18.86	3.55	7.00	16.50	19.00	21.00	29.00	
Rehabilitation	78	19.56	3.51	9.92	17.50	19.92	21.50	29.00	
Community	35	19.47	3.85	11.67	15.58	19.58	22.83	27.67	
Professional category									
Specialist nurse	151	19.32	3.47	8.17	17.42	19.33	21.25	29.00	**0.0058** *
Nurse	526	18.52	3.41	7.00	16.33	18.42	20.50	30.00	
Work department									
Medicine	223	19.31	3.40	10.17	17.00	19.25	21.50	29.08	**0.0104** **
Surgery	203	18.34	3.82	7.00	16.00	18.17	20.25	30.00	
Intensive Care	113	18.96	2.89	11.83	17.33	19.17	20.50	26.50	
Emergency	68	18.26	3.10	13.17	16.08	18.00	19.92	27.17	
Woman and Child	77	18.20	3.49	8.17	15.92	18.83	20.17	27.67	
Work schedule									
Day shifts	161	19.84	3.69	7.00	17.50	19.58	22.00	29.00	**<0.0001** *
Day and night shifts	523	19.39	3.31	7.00	16.17	18.33	20.33	30.00	

*n*—absolute frequency; S.D.—Standard deviation; Min.—Minimum; Q1—Quartile 1; Q3—Quartile 3; Max.—Maximum. * *p*-value obtained using the Mann–Whitney U test. ** *p*-value obtained using the Kruskal–Wallis test.

**Table 4:** Results of Dunn’s post-hoc test for pairwise comparisons of structural empowerment between groups.

Variable	Pairwise Comparisons	*p*-Value
CWEQ–II Total Score	Generation	
	Generation Z × Generation Y or Millennials	**0.004**
	Generation Z × Generation X	0.472
	Generation Y or Millennials × Generation X	0.099
CWEQ–II Total Score	Work Department	
	Medicine × Surgery	**0.039**
	Medicine × Intensive Care	1.000
	Medicine × Emergency	0.129
	Medicine × Woman and Child	0.381
	Surgery × Intensive Care	0.572
	Surgery × Emergency	1.000
	Surgery × Woman and Child	1.000
	Intensive Care × Emergency	0.612
	Intensive Care × Woman and Child	1.000
	Emergency × Woman and Child	1.000

## Data Availability

The data that support the findings of this study are available from the corresponding author upon reasonable request.

## Public Involvement Statement

No public involvement in any aspect of this research.

## Guidelines and Standards Statement

This manuscript was drafted against the STROBE (Strengthening the Reporting of Observational Studies in Epidemiology) for observational studies (descriptive correlational).

## Abbreviations

The following abbreviations are used in this manuscript:

CWEQ–II	Conditions of Work Effectiveness Questionnaire II
GE	Global Empowerment
ICN	International Council of Nurses
SD	Standard deviation

## References

[1] Pereira S., Ribeiro M., Mendes M., Ferreira R., Santos E., Fassarella C., Ribeiro O. (2024). Positive Nursing Practice Environment: A Concept Analysis. Nurs. Rep..

[2] International Council of Nurses (2025). Caring for Nurses Strengthens Economies. Our Nurses. Our Future.

[3] Boudreau C., Rhéaume A. (2024). Impact of the Work Environment on Nurse Outcomes: A Mediation Analysis. West. J. Nurs. Res..

[4] Jun J., Ojemeni M.M., Kalamani R., Tong J., Crecelius M.L. (2021). Relationship between nurse burnout, patient and organizational outcomes: Systematic review. Int. J. Nurs. Stud..

[5] Mabona J.F., van Rooyen D., Ham-Baloyi W.T. (2022). Best practice recommendations for healthy work environments for nurses: An integrative literature review. Health SA Gesondheid.

[6] Ribeiro O.M.P.L., Coimbra V.M.O., Pereira S.C.A., Faria A.C.A., Teles P.J.F.C., Rocha C.G. (2022). Impact of COVID-19 on the Environments of Professional Nursing Practice and Nurses’ Job Satisfaction. Int. J. Environ. Res. Public Health.

[7] Alanazi F.K., Lapkin S., Molloy L., Sim J. (2023). The impact of safety culture, quality of care, missed care and nurse staffing on patient falls: A multisource association study. J. Clin. Nurs..

[8] Eva G.F., Amo-Setién F., César L.C., Concepción S.S., Roberto M.M., Jesús M.M., Carmen O.M. (2024). Effectiveness of intervention programs aimed at improving the nursing work environment: A systematic review. Int. Nurs. Rev..

[9] Lee S.A., Lee J. (2023). Differences in occupational values, communication types, job satisfaction, and organisational commitment among clinical nurses across generations. Front. Psychol..

[10] Pereira S.C.A., Ribeiro O.M.P.L., Fassarella C.S., Santos E.J.F. (2023). The impact of nursing practice environments on patient safety culture in primary health care: A scoping review protocol. BJGP Open.

[11] Almeida S., Nascimento A., Lucas P.B., Jesus É., Araújo B. (2020). RN4CAST Study in Portugal: Validation of the Portuguese Version of the Practice Environment Scale of the Nursing Work Index. Aquichan.

[12] Ribeiro O.M.P.L., Trindade L.L., Fassarella C.S., Pereira S.C.A., Teles P.J.F.C., Rocha C.G., Leite P.C.S., Ventura-Silva J.M.A., Sousa C.N. (2022). Impact of COVID-19 on professional nursing practice environments and patient safety culture. J. Nurs. Manag..

[13] Abuzied Y., Al-Amer R., Abuzaid M., Somduth S. (2022). The Magnet Recognition Program and Quality Improvement in Nursing. Glob. J. Qual. Saf. Healthc..

[14] Travis A., Fitzpatrick J.J. (2025). Examining the Relationship Between Hospital Nurses’ Structural Empowerment, Missed Nursing Care and Quality of Care: A Cross-Sectional Study. J. Clin. Nurs..

[15] Kanter R.M. (1981). Power, leadership, and participatory management. Theory Pract..

[16] Fragkos K.C., Makrykosta P., Frangos C.C. (2020). Structural empowerment is a strong predictor of organizational commitment in nurses: A systematic review and meta-analysis. J. Adv. Nurs..

[17] Moura A.A., Souza A.A.C.F., Silva P.K.A., Bernardes A., Ferreira N.P. (2024). Empoderamento estrutural de enfermeiros nos serviços de emergências: Revisão integrativa. Acta Paul. Enferm..

[18] Laschinger H.K.S. (2012). Conditions for Work Effectiveness Questionnaire I and II: User Manual.

[19] Choi S., Kim M. (2019). Effects of structural empowerment and professional governance on autonomy and job satisfaction of the Korean nurses. J. Nurs. Manag..

[20] Lu H., Zhao Y., While A. (2019). Job satisfaction among hospital nurses: A literature review. Int. J. Nurs. Stud..

[21] Orgambídez A., Almeida H. (2020). Exploring the link between structural empowerment and job satisfaction through the mediating effect of role stress: A cross-sectional questionnaire study. Int. J. Nurs. Stud..

[22] Teixeira A.C., Barbieri-Figueiredo M.C. (2015). Nursing empowerment and job satisfaction: An integrative review according the Structural Theory. Rev. Enf. Ref..

[23] Narzary G., Palo S. (2020). Structural empowerment and organisational citizenship behaviour: The mediating–moderating effect of job satisfaction. Pers. Rev..

[24] Rawah R., Banakhar M. (2022). The Relationship between Empowerment and Organizational Commitment from Nurse’s Perspective in the Ministry of Health Hospitals. Healthcare.

[25] Singh M.D., Pilkington F.B., Patrick L. (2014). Empowerment and mentoring in nursing academia. Int. J. Nurs. Educ. Sch..

[26] Yang J., Liu Y., Chen Y., Pan X. (2014). The effect of structural empowerment and organizational commitment on Chinese nurses’ job satisfaction. Appl. Nurs. Res..

[27] Çelik Ş.S., Sariköse S., Çelik Y. (2024). Structural and psychological empowerment and burnout among nurses: A systematic review and meta-analysis. Int. Nurs. Rev..

[28] Newberry L.W. (2021). Using Structural Empowerment to Improve Outcomes. J. Nurs. Adm..

[29] Alenazi L. (2023). Nursing structural empowerment: A concept analysis. Nurse Health J. Keperawatan.

[30] Wang Z., Yang L., Zhu Y., Tang X., Wang T., Chen L., Li L., Xie W., Peng J., Yang J. (2024). Innovative behavior and structural empowerment among the Chinese clinical nurses: The mediating role of decent work perception. BMC Nurs..

[31] Moura L.N., Camponogara S., Santos J.L.G.D., Gasparino R.C., Silva R.M.D., Freitas E.O. (2020). Structural empowerment of nurses in the hospital setting. Rev. Lat. Am. Enferm..

[32] von Elm E., Altman D.G., Egger M., Pocock S.J., Gøtzsche P.C., Vandenbroucke J.P., STROBE Initiative (2007). The Strengthening the Reporting of Observational Studies in Epidemiology (STROBE) statement: Guidelines for reporting observational studies. Lancet.

[33] Centro Hospitalar Universitário São João (2024). Relatório & Contas 2023.

[34] Teixeira A.C., Nogueira M., Alves P. (2016). Structural empowerment in nursing: Translation, adaptation and validation of the Conditions of Work Effectiveness Questionnaire II. Rev. Enferm. Ref..

[35] Laschinger H.K., Finegan J., Shamian J., Wilk P. (2001). Impact of structural and psychological empowerment on job strain in nursing work settings: Expanding Kanter’s model. J. Nurs. Adm..

[36] Pagano M., Gauvreau K., Mattie H. (2022). Principles of Biostatistics.

[37] Cohen J. (1992). A power primer. Psychol. Bull..

[38] Cronbach L.J. (1951). Coefficient Alpha and the Internal Structure of Tests. Psychometrika.

[39] Prinsen C.A.C., Mokkink L.B., Bouter L.M., Alonso J., Patrick D.L., de Vet H.C.W., Terwee C.B. (2018). COSMIN guideline for systematic reviews of patient-reported outcome measures. Qual. Life Res..

[40] Ordem dos Enfermeiros (2024). Estatística de Enfermeiros.

[41] Teixeira A.C., Nogueira A., Nunes J.R., Teixeira L., Barbieri-Figueiredo M.C. (2021). Professional empowerment among Portuguese nursing staff: A correlational study. J. Nurs. Manag..

[42] Ribeiro O.M.P.L., Cardoso M.F., Trindade L.L., Rocha C.G., Teles P.J.F.C., Pereira S., Coimbra V., Ribeiro M.P., Reis A., Faria A.C.A. (2023). From the first to the fourth critical period of COVID-19: What has changed in nursing practice environments in hospital settings?. BMC Nurs..

[43] Yesilbas H., Kantek F. (2024). Relationship between structural empowerment and job satisfaction among nurses: A meta-analysis. Int. Nurs. Rev..

[44] Al-Ghwary A.A., Al-Oweidat I.A., Al-Qudimat A.R., Abu Shosha G.M., Khalifeh A.H., ALBashtawy M. (2024). The Impact of Work Environment on Structural Empowerment among Nurses in Governmental Hospitals. Nurs. Rep..

[45] Sanches D., Pereira S., Castro S., Mendes M., Santos E., Ribeiro O. (2024). Generational diversity in nursing practice environments-scoping review. BMC Nurs..

[46] Pawlak N., Serafin L., Czarkowska-Pączek B. (2022). Analysis of the influence of intergenerational differences on cross-generational cooperation among nurses. Nurs. 21st Century.

[47] Stevanin S., Palese A., Bressan V., Vehviläinen-Julkunen K., Kvist T. (2018). Workplace-related generational characteristics of nurses: A mixed-method systematic review. J. Adv. Nurs..

[48] Moon Y., Lee S. (2025). Experiences of Generation Z Nurses Adapting to Work in a Tertiary Hospital: A Grounded Theory Study. J. Adv. Nurs..

[49] White J., Hepworth G., Alvorado J., Lemmon C., Brijnath B. (2021). Managing workplace change: Intergenerational perspectives from Victorian public hospital nurses. Collegian.

[50] Balay-odao E.M., Cruz J.P., Alquwez N., Al Otaibi K., Al Thobaity A., Alotaibi R.S., Valencia J.A., Danglipen C.C. (2022). Structural empowerment and work ethics influence on the work engagement of millennial nurses. J. Nurs. Manag..

[51] Al Sabei S., Labrague L., Cayaban A., Al-Rawjafah O., Burney I., AbulRub R. (2025). Emotional exhaustion among critical care nurses and its link to occupational stress, structural empowerment, and perceived work environment: Is there a generational difference?. J. Intensive Care Soc..

[52] Ordem dos Enfermeiros (2025). Regulamento n.º 395/2025: Regulamento das Especialidades e Competências Acrescidas da Ordem dos Enfermeiros.

[53] Kanter R.M. (1993). Men and Women of the Corporation.

[54] Laschinger H.K., Wong C.A., Cummings G.G., Grau A.L. (2014). Resonant leadership and workplace empowerment: The value of positive organizational cultures in reducing workplace incivility. Nurs. Econ..

[55] Ruano-Ferrer F., Gutiérrez-Giner M.I. (2023). Safety perception in the operating environment: The nurses’ perspective versus that of the surgeons. Heliyon.

[56] Mrayyan M.T., Abu Khait A., Rababa M., Algunmeeyn A., Al-Rawashdeh S., Al-Atiyyat N., Rababa M., Abu Saraya A., Al-Rjoub S. (2024). Professional autonomy in nursing: A concept analysis. SAGE Open.

